# Quality of life and social support as key determinants of anxiety and depression in Myasthenia Gravis: evidence from a Chinese cohort

**DOI:** 10.3389/fneur.2025.1670436

**Published:** 2025-11-12

**Authors:** Xiang Li, Zhipeng Li, Chaoyue Zhang, Long Li, Yimin Liang, Xuxiang Zhang, Fengbin Liu, Xinfeng Lin, Qilong Jiang

**Affiliations:** 1 The First Clinical Medical College of Guangzhou University of Chinese Medicine, Guangzhou, Guangdong, China; 2 The First Affiliated Hospital of Guangzhou University of Chinese Medicine, Guangzhou, Guangdong, China

**Keywords:** Myasthenia Gravis, anxiety, depression, disease severity, quality of life, social support

## Abstract

**Background:**

Myasthenia Gravis (MG) is a chronic autoimmune disorder frequently accompanied by anxiety and depression, which aggravate disease burden. Evidence on the relationship between clinical characteristics and psychological symptoms in MG remains inconsistent.

**Methods:**

We conducted a cross-sectional study of 93 Chinese MG patients. Clinical and demographic data were collected, and anxiety and depression were assessed using the Hospital Anxiety and Depression Scale (HADS). Associations were examined by correlation, chi-square tests, and logistic regression with false discovery rate (FDR) correction. Multivariable models adjusted for age and sex. ROC curve analyses evaluated the predictive performance of (MG Activities of Daily Living) MG-ADL, (15-item Myasthenia Gravis Quality of Life scale) MG-QOL-15, and (Social Support Rating Scale) SSRS.

**Results:**

Anxiety and depression were present in 30.1 and 35.5% of patients, respectively. In multivariable analysis, reduced quality of life (MG-QOL-15, OR = 0.85, 95% CI: 0.78–0.92, *p* < 0.001) and insufficient social support (SSRS, OR = 1.08, 95% CI: 1.00–1.16, *p* = 0.03) independently predicted psychological distress, whereas MGFA classification was not significant. Supplementary linear regression confirmed these findings. ROC analysis showed MG-QOL-15 had the best performance in detecting both anxiety (AUC = 0.884) and depression (AUC = 0.837), while SSRS had moderate value and MG-ADL limited discrimination.

**Conclusion:**

Psychological distress in MG is more closely linked to quality of life and social support than to conventional disease severity. Routine psychological screening with MG-QOL-15 and SSRS may facilitate early identification of high-risk patients.

## 1 Introduction

Myasthenia Gravis (MG) is a rare chronic autoimmune disorder primarily affecting the neuromuscular junction. The core pathophysiology of MG involves aberrant immune responses, wherein autoantibodies targeting acetylcholine receptors (AChR) or muscle-specific kinase (MuSK) disrupt neuromuscular transmission. This results in fluctuating skeletal muscle weakness and fatigue, often exacerbated by exertion and partially alleviated by rest (1). Clinically, MG manifestations range from mild ocular symptoms to generalized weakness, with severe cases potentially compromising respiratory muscles and posing life-threatening risks.

Although MG has a relatively low incidence, its prevalence has increased in recent years, likely due to improved diagnostic capabilities and heightened clinical awareness (2). The immunopathogenesis of MG is multifactorial; while the presence of AChR antibodies is a hallmark, other immune components—such as the complement system, B cells, and T cells—are also implicated (3, 4). Accordingly, treatment strategies focus on immunomodulatory approaches, including immunosuppressive agents, cholinesterase inhibitors, and therapeutic plasma exchange.

In addition to its somatic symptoms, MG is frequently accompanied by psychological disturbances. A growing body of evidence indicates that anxiety and depression are among the most prevalent psychiatric comorbidities in patients with MG. These mental health conditions can significantly impair quality of life and may also negatively influence disease progression by affecting treatment adherence and modulating immune function. Symptoms such as persistent low mood, heightened tension, insomnia, and concentration difficulties are commonly reported (5–7). The prevalence of anxiety and depression in MG patients ranges from 33 to 36%, with variations likely due to differences in sample characteristics, disease severity, and study methodology (8).

The link between mental health and autoimmune diseases has garnered increasing scientific interest. Anxiety and depression are not only common psychological burdens but may also contribute to disease progression through complex biopsychosocial mechanisms (9, 10). In MG patients, physical debilitation, the chronic nature of the disease, and uncertainties surrounding treatment outcomes may exacerbate psychological distress. Conversely, psychological stress may aggravate disease severity by suppressing immune function or enhancing inflammatory responses (11, 12). While several studies have identified a positive correlation between disease severity and psychiatric symptoms in MG, findings remain inconsistent (5). These discrepancies may be attributed to differences in study design, population heterogeneity, or variability in psychological assessment tools.

Recent evidence also points to additional factors influencing psychiatric comorbidities in MG. Seronegative MG poses unique diagnostic and therapeutic challenges, often resulting in delays and uncertainty that may exacerbate psychological distress (13). Moreover, sex-related differences in neuromuscular diseases, including MG, have been documented, with biological and psychosocial factors potentially contributing to differential vulnerability to anxiety and depression (14). These perspectives underscore the heterogeneity of MG populations and further justify the need for comprehensive evaluation of psychiatric burden.

Against this background, the primary aim of the present study is to investigate the associations of anxiety and depressive symptoms with clinical characteristics in patients with MG. In addition to examining traditional indicators of disease severity, we also focused on quality of life and social support, which have been increasingly recognized as critical determinants of psychological well-being. By integrating these multidimensional factors, this study seeks to provide a more comprehensive understanding of the psychiatric burden in MG.

Furthermore, unlike most previous studies conducted in Western populations, our work focuses on a Chinese cohort, where cultural context and social support structures may shape psychological outcomes differently. By employing multiple validated instruments and establishing ROC-derived cut-off thresholds, we aimed to generate clinically applicable criteria that can facilitate early psychiatric screening and inform targeted interventions in MG patients.

## 2 Methods

### 2.1 Study design

This single-center, cross-sectional study was conducted to investigate the relationship between anxiety and depressive symptoms and disease severity in patients with MG. The study was carried out at the First Affiliated Hospital of Guangzhou University of Chinese Medicine between October and December 2024. The study has been approved by the Ethics Committee of the First Affiliated Hospital of Guangzhou University of Chinese Medicine (K-2024-163). All participants provided written informed consent prior to participation. The research follows the ethical principles outlined in the Declaration of Helsinki, with all patients being informed and voluntarily participating. A total of 93 patients diagnosed with MG according to the Chinese Guidelines for the Diagnosis and Treatment of Myasthenia Gravis (2020) were enrolled (15). All participants provided written informed consent prior to participation. Inclusion criteria were as follows: patients aged between 18 and 70 years, with a confirmed diagnosis of MG, and the cognitive ability to understand and complete study questionnaires. Patients were excluded if they had severe cognitive impairment, significant communication difficulties, or declined to participate.

Comprehensive data collection was performed to capture both clinical and psychological profiles. Demographic variables included gender, age, marital status, educational attainment, and household income, to explore potential associations with anxiety and depression. Clinical characteristics encompassed disease onset, duration, (Myasthenia Gravis Foundation of America) MGFA classification, AChR antibody status, presence of thymoma, and history of myasthenic crisis, including frequency of crises.

Detailed treatment histories were also recorded, with particular focus on medications such as corticosteroids and immunosuppressants, as these may influence psychological health. Disease severity was primarily assessed using the MGFA classification system, ranging from Class I (ocular involvement only) to Class V (most severe, requiring ventilatory support) (16). Additionally, the MG Activities of Daily Living (MG-ADL) scale was used to evaluate the impact of symptoms on daily functioning across ocular, bulbar, respiratory, and limb domains, with higher scores indicating greater severity (17).

Psychological symptoms were assessed using the Hospital Anxiety and Depression Scale (HADS), which comprises two subscales: HADS-A (anxiety) and HADS-D (depression). Each subscale consists of seven items scored from 0 to 3. Total scores of 0–7 are considered normal, 8–10 suggest mild symptoms, 11–14 indicate moderate symptoms, and 15–21 reflect severe anxiety or depression (18). HADS is a well-validated tool widely used in clinical populations to detect anxiety and depression. Numerous studies conducted across diverse populations have validated the Hospital Anxiety and Depression Scale (HADS) as a reliable and effective instrument for assessing anxiety and depression (19–22).

To evaluate social support, the Social Support Rating Scale (SSRS) was administered. Developed by Xiao Shuiyuan to suit the Chinese sociocultural context, the SSRS has demonstrated good reliability and validity (23, 24). It includes 10 items covering three dimensions: subjective support, objective support, and support utilization. Total scores above 44 indicate high social support, scores between 23 and 44 represent moderate support, and scores below 23 reflect low support levels. Social support is recognized as a critical determinant of mental health, with low levels associated with an increased risk of anxiety and depression.

Patients’ quality of life was assessed using the 15-item Myasthenia Gravis Quality of Life scale (MG-QOL-15), which measures the impact of MG on various aspects of daily living (25). Lower scores indicate more profound reductions in quality of life. All patients underwent assessment with these scales upon enrollment to evaluate their psychological health status and disease condition. All data were collected by rigorously trained research personnel to ensure accuracy and consistency.

### 2.2 Statistical analysis

Statistical analyses were performed using SPSS (version 27, IBM Corp., Armonk, NY). Two-tailed *p* < 0.05 was considered statistically significant.

Spearman’s correlation coefficients were first calculated to examine associations between HADS scores and clinical variables. Univariate logistic regression analyses were then conducted to identify potential predictors of anxiety and depression, with results expressed as odds ratios (ORs) and 95% confidence intervals (CIs). To control for multiple testing, the Benjamini–Hochberg false discovery rate (FDR) correction was applied. Variables with *p* < 0.10 in univariate analyses were subsequently entered into multivariable logistic regression models, with additional adjustment for age and sex. The MGFA classification was also retained in multivariable analyses given its clinical relevance as an indicator of disease severity, despite not reaching significance in univariate models.

Receiver Operating Characteristic (ROC) curves were constructed to assess the predictive performance of the MG-ADL, MG-QOL-15, and SSRS in identifying anxiety and depression. The area under the curve (AUC) and 95% CIs were calculated using DeLong’s method (26). Optimal cut-off values were determined by the Youden index, and corresponding sensitivity and specificity values were reported.

Finally, multiple linear regression was conducted as a supplementary analysis, treating HADS-A and HADS-D scores as continuous outcomes. Unstandardized regression coefficients (B) with 95% CIs were reported to facilitate clinical interpretation. This approach served as a robustness check to confirm whether the observed associations persisted when symptom severity was modeled on a continuous scale.

## 3 Results

### 3.1 Baseline characteristics and correlation analysis of anxiety and depression in MG patients

A total of 93 patients with a confirmed diagnosis of MG were included in the study (Tables 1, 2). Among them, 28 patients (30.1%) exhibited symptoms of anxiety, while 65 patients (69.9%) did not. Additionally, 33 patients (35.5%) presented with depressive symptoms, whereas 60 patients (64.5%) did not. The mean age of participants was 47.26 ± 15.05 years. Females accounted for 69.9% (65/93) of the cohort, and males for 30.1% (28/93). The vast majority were of Han ethnicity (98.9%). Regarding socioeconomic status, 60.6% (43/93) had completed at least a high school education, 80.6% (75/93) were married, and 81.5% (75/93) had children.

**Table 1:** Correlations of MG patients’ clinical factors with anxiety HADS score.

Clinical Factors	Anxiety (*N* = 28)	Non-anxiety (*N* = 65)	Total	Correlation (95% CI)	*p*-value
*Age (years)*	47.75 ± 15.88	47.05 ± 14.80	47.26 ± 15.05	0.01 (−0.20 ~ 0.22)	0.960
*Gender*				−0.09 (−0.29 ~ 0.12)	0.404
Male	10 (35.7)	18 (27.7)	28 (30.1)		
Female	18 (64.3)	47 (72.3)	65 (69.9)		
*Nationality*				0.08 (−0.13 ~ 0.29)	0.442
Han	27 (96.4)	61 (100.0)	91 (98.9)		
Others	1 (3.6)	0	1 (1.1)		
*High school or above*				0.11 (−0.14 ~ 0.34)	0.380
Yes	10 (47.6)	33 (66.0)	43 (60.6)		
No	11 (52.4)	17 (34.0)	28 (39.4)		
*Married*				−0.11 (−0.31 ~ 0.10)	0.286
Yes	24 (85.7)	51 (78.5)	75 (80.6)		
No	4 (14.3)	14 (21.5)	18 (19.4)		
*Have children*				−0.12 (−0.32 ~ 0.10)	0.260
Yes	24 (85.7)	51 (79.7)	75 (81.5)		
No	4 (14.3)	13 (20.3)	17 (18.5)		
*Separated*				−0.10 (−0.31 ~ 0.11)	0.336
Yes	5 (18.5)	5 (7.8)	10 (11.0)		
No	22 (81.5)	59 (92.2)	81 (89.0)		
*MGFA Clinical Classification*				0.32(0.12 ~ 0.50)	0.002*
I	3 (10.7)	14 (21.5)	17 (18.3)		
II	12 (42.9)	39 (60.0)	51 (54.8)		
III	7 (25.0)	7 (10.8)	14 (15.1)		
IV–V	6 (21.4)	5 (7.7)	11 (11.8)		
*Antibodies [AChR-Ab (+)]*				−0.07 (−0.30 ~ 0.16)	0.535
Yes	20 (80.0)	42 (80.8)	62 (80.5)		
No	5 (20.0)	10 (19.2)	15 (19.5)		
*Thymic Condition (Thymoma)*				−0.07 (−0.28 ~ 0.15)	0.548
Yes	14 (51.9)	29 (48.3)	43 (49.4)		
No	13 (48.1)	31 (51.7)	44 (50.6)		
*Time since onset (years)*					
	6.39 ± 5.99	7.03 ± 7.47	6.84 ± 7.03	0.01 (−0.21 ~ 0.21)	0.984
*Ever glucocorticoid use*				−0.19 (−0.38 ~ 0.02)	0.072
Yes	26 (92.9)	57 (87.7)	83 (89.2)		
No	2 (7.1)	8 (12.3)	10 (10.8)		
*Ever immunosuppresant use (except glucocorticoid)*				−0.08 (−0.28 ~ 0.14)	0.466
Yes	18 (64.3)	36 (55.4)	54 (58.1)		
No	10 (35.7)	29 (44.6)	39 (41.9)		
*Combined with other diseases*				−0.24 (−0.43 ~ −0.03)	0.020*
Yes	22 (78.6)	33 (50.8)	55 (59.1)		
No	6 (21.4)	32 (49.2)	38 (40.9)		
*Number of MG Crisis*					
	1.26 ± 1.46	0.83 ± 1.55	0.96 ± 1.53	0.25 (0.04 ~ 0.44)	0.016*
*MG-ADL score*	6.00 ± 4.77	3.12 ± 2.88	3.99 ± 3.77	0.38 (0.18 ~ 0.55)	<0.001*
*MG-QOL-15 score*					
	30.29 ± 9.59	14.15 ± 10.05	19.01 ± 12.36	0.74 (0.63 ~ 0.82)	<0.001*
*SSRS score*	38.11 ± 7.20	43.72 ± 8.62	42.03 ± 8.58	−0.35 (−0.52 ~ −0.15)	<0.001*

**Table 2:** Correlations of MG patients’ clinical factors with depression HADS score.

Clinical Factors	Depression (*N* = 33)	Non-depression (*N* = 60)	Total	Correlation (95% CI)	*p*-value
*Age (years)*	48.45 ± 15.22	46.60 ± 15.04	47.26 ± 15.05	0.05 (−0.17 ~ 0.25)	0.667
*Gender*				−0.06 (−0.26 ~ 0.16)	0.596
Male	9 (27.3)	19 (31.7)	28 (30.1)		
Female	24 (72.7)	41 (68.3)	65 (69.9)		
*Nationality*				0.07 (−0.14 ~ 0.28)	0.511
Han	32 (97.0)	59 (100.0)	91 (98.9)		
Others	1 (3.0)	0	1 (1.1)		
*High school or above*				0.17 (−0.08 ~ 0.39)	0.161
Yes	13 (52.0)	30 (65.2)	43 (60.6)		
No	12 (48.0)	16 (34.8)	28 (39.4)		
*Married*				−0.07 (−0.28 ~ 0.14)	0.498
Yes	28 (84.8)	47 (78.3)	75 (80.6)		
No	5 (15.2)	13 (21.7)	18 (19.4)		
*Have children*				−0.06 (−0.26 ~ 0.16)	0.595
Yes	28 (84.8)	47 (79.7)	75 (81.5)		
No	5 (15.2)	12 (20.3)	17 (18.5)		
*Separated*				−0.08 (−0.28 ~ 0.14)	0.475
Yes	5 (15.6)	5 (8.5)	10 (11.0)		
No	27 (84.4)	54 (91.5)	81 (89.0)		
*MGFA Clinical Classification*				0.31 (0.11 ~ 0.49)	0.003*
I	4 (12.1)	13 (21.7)	17 (18.3)		
II	15 (45.5)	36 (60.0)	51 (54.8)		
III	8 (24.2)	6 (10.0)	14 (15.1)		
IV–V	6 (18.2)	5 (8.3)	11 (11.8)		
*Antibodies [AChR-Ab (+)]*				−0.08 (−0.30 ~ 0.16)	0.510
Yes	23 (79.3)	39 (81.3)	62 (80.5)		
No	6 (20.7)	9 (18.8)	15 (19.5)		
*Thymic Condition (Thymoma)*				−0.06 (−0.27 ~ 0.16)	0.615
Yes	14 (45.2)	29 (51.8)	43 (49.4)		
No	17 (54.8)	27 (48.2)	44 (50.6)		
*Time since onset (years)*					
	5.96 ± 6.66	7.32 ± 7.23	6.84 ± 7.03	−0.03 (−0.24 ~ 0.18)	0.798
*Ever glucocorticoid use*				−0.20 (−0.40 ~ 0.01)	0.049*
Yes	31 (93.9)	52 (86.7)	83 (89.2)		
No	2 (6.1)	8 (13.3)	10 (10.8)		
*Ever immunosuppresant use (except glucocorticoid)*				−0.19 (−0.38 ~ 0.03)	0.076
Yes	23 (69.7)	31 (51.7)	54 (58.1)		
No	10 (30.3)	29 (48.3)	39 (41.9)		
*Combined with other diseases*				−0.31 (−0.48 ~ −0.10)	0.003*
Yes	23 (69.7)	32 (53.3)	55 (59.1)		
No	10 (30.3)	28 (46.7)	38 (40.9)		
*Number of MG Crisis*				0.25 (0.04 ~ 0.44)	0.016*
	1.22 ± 1.43	0.82 ± 1.57	0.96 ± 1.53		
*MG-ADL score*	5.45 ± 4.89	3.18 ± 2.71	3.99 ± 3.77	0.26 (0.05 ~ 0.45)	0.012*
*MG-QOL-15 score*					
	28.03 ± 10.62	14.05 ± 10.31	19.01 ± 12.36	0.68 (0.55 ~ 0.78)	<0.001*
*SSRS score*	39.55 ± 7.18	43.40 ± 9.03	42.03 ± 8.58	−0.36 (−0.53 ~ −0.16)	<0.001*

Data are presented as *n* (%), mean ± SD.

^*^p < 0.05 is significant.

MG, myasthenia gravis; HADS, Hospital Anxiety and Depression Scale.

AChR acetylcholine receptor, MGFA Myasthenia Gravis Foundation of America, MG-ADL Myasthenia Gravis Activities of Daily Living, MG-QOL-15 Myasthenia Gravis Quality of Life 15-item scale, SSRS Social Support Rating Scale.

In terms of clinical classification based on the MGFA system, most patients were classified as Class II (54.8%) or Class I (18.3%), while fewer were classified as Class III (15.1%) or Classes IV–V (11.8%). AChR antibodies were positive in 80.5% (62/93) of patients, and 49.4% (43/93) had comorbid thymoma. The mean disease duration was 6.84 ± 7.03 years. With respect to treatment, 89.2% (83/93) had received corticosteroids, and 58.1% (54/93) had been treated with immunosuppressants. Additionally, 59.1% (55/93) had comorbid conditions, and 10.8% (10/93) had experienced at least one myasthenic crisis.

Correlation analyses revealed that MGFA classification was significantly positively associated with anxiety (*r* = 0.32, *p* = 0.002) and depression (*r* = 0.31, *p* = 0.003), indicating that more severe disease was associated with higher rates of psychological distress. The frequency of myasthenic crises was also significantly associated with both anxiety (*r* = 0.25, *p* = 0.016) and depression (*r* = 0.25, *p* = 0.016), suggesting that crisis experiences may exacerbate psychological burden.

The MG-ADL score, which reflects the impact of disease on daily functioning, was positively correlated with anxiety (*r* = 0.38, *p* < 0.001) and depression (*r* = 0.26, *p* = 0.012), indicating that greater functional impairment was linked to worse psychological outcomes. MG-QOL-15 scores showed a strong positive correlation with anxiety (*r* = 0.74, *p* < 0.001) and depression (*r* = 0.68, *p* < 0.001), underscoring the substantial influence of diminished quality of life on mental health.

Furthermore, the SSRS score was negatively correlated with anxiety (*r* = −0.35, *p* < 0.001) and depression (*r* = −0.36, *p* < 0.001), suggesting that lower levels of social support were associated with increased psychological distress. Notably, patients with comorbid conditions had higher levels of anxiety (*r* = −0.24, *p* = 0.020) and depression (*r* = −0.31, *p* = 0.003), potentially due to the compounded burden of multiple health issues. In addition, the use of corticosteroids was significantly associated with higher rates of depression (*r* = −0.20, *p* = 0.049), although no significant association was observed with anxiety (*p* = 0.072), implying that corticosteroids may influence mood disorders.

By contrast, factors such as age, gender, education level, marital status, AChR antibody positivity, presence of thymoma, disease onset age, and use of immunosuppressants did not show statistically significant correlations with either anxiety or depression (*p* > 0.05).

### 3.2 Association between MG disease severity and symptoms of anxiety and depression

**Figure 1:**
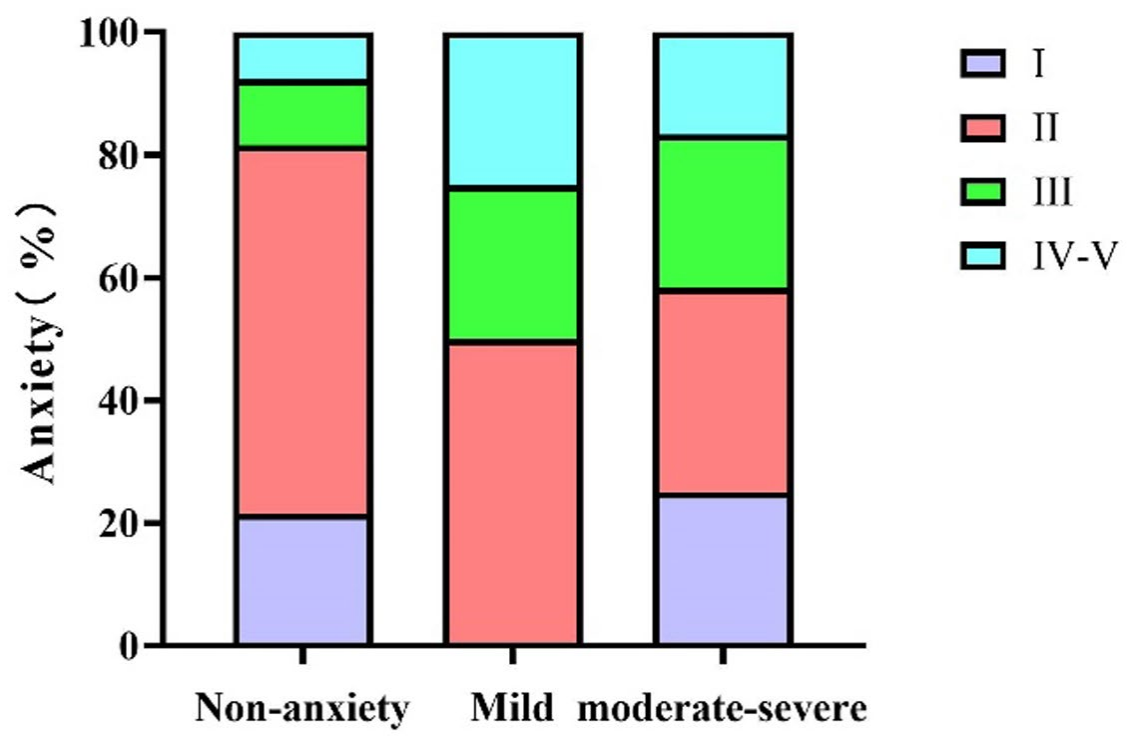
The relationship between MG disease severity and anxiety in MG patients. MG, Myasthenia Gravis.

**Figure 2:**
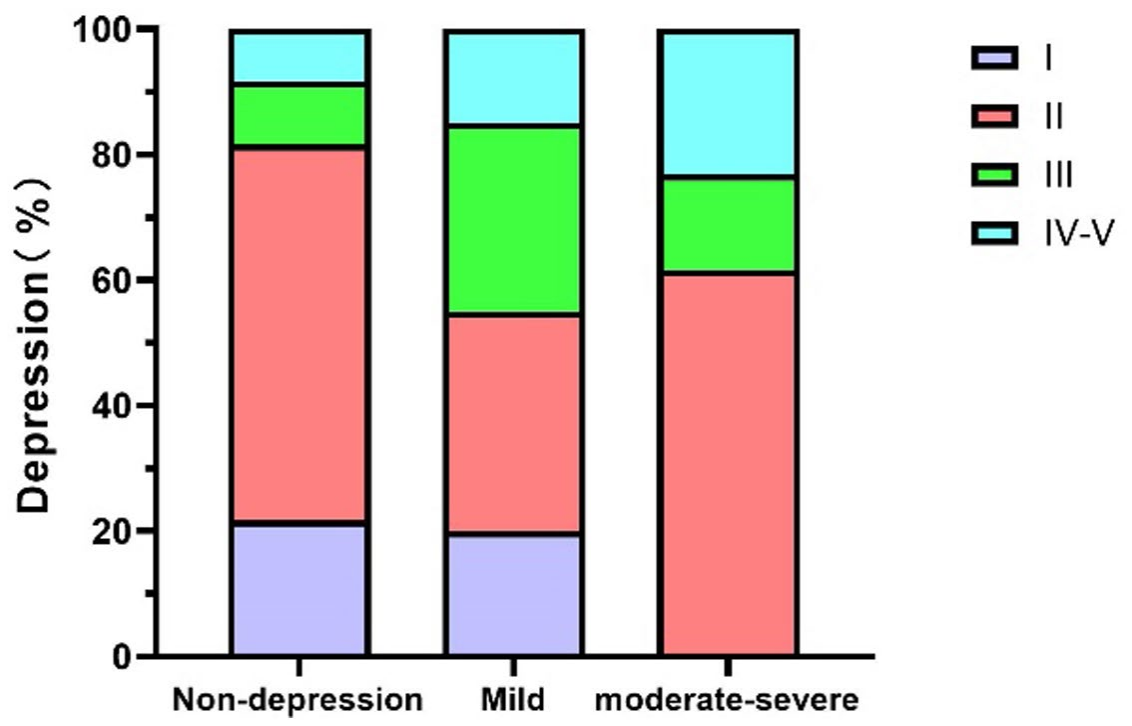
The relationship between MG disease severity and depression in MG patients. MG, Myasthenia Gravis.

Table 3 (corresponding to Figure 1), Table 4 (corresponding to Figure 2) further explore the relationship between the severity of MG disease and the presence of anxiety and depression symptoms. Chi-square tests were used to assess the distribution of anxiety and depression symptoms across different MGFA classification levels. The results indicate a clear upward trend in the prevalence of both anxiety and depression with increasing disease severity.

**Table 3:** The relationship between MG disease severity and anxiety in MG patients.

Disease severity	Non-anxiety (*N* = 65)	Anxiety		Total (*N* = 93)	*X* ^2^	*p* value
		Mild (*N* = 16)	Moderate–severe (*N* = 12)			
I	14 (21.5)	0	3 (25.0)	17 (18.3)	12.25	0.032
II	39 (60.0)	8 (50.0)	4 (33.3)	51 (54.8)		
III	7 (10.8)	4 (25.0)	3 (25.0)	14 (15.1)		
IV–V	5 (7.7)	4 (25.0)	2 (16.7)	11 (11.8)		

**Table 4:** The relationship between MG disease severity and depression in MG patients.

Disease severity	Non-depression (*N* = 60)	Depression		Total (*N* = 93)	*X* ^2^	*p* value
		Mild (*N* = 20)	Moderate–severe (*N* = 13)			
I	13 (21.7)	4 (20.0)	0	17 (18.3)	11.054	0.061
II	36 (60.0)	7 (35.0)	8 (61.5)	51 (54.8)		
III	6 (10.0)	6 (30.0)	2 (15.4)	14 (15.1)		
IV–V	5 (8.3)	3 (15.0)	3 (23.1)	11 (11.8)		

^*^p < 0.05 is significant. MG, Myasthenia Gravis.

As shown in Table 3 (corresponding to Figure 1), there was a statistically significant association between MGFA classification and the occurrence of anxiety symptoms (*χ*^2^ = 12.25, *p* = 0.032). In the MGFA Class I group, 76.5% of patients had no anxiety symptoms, while 23.5% reported having anxiety symptoms. In the MGFA Class II group, 29.4% of patients presented with anxiety symptoms, with 50% of these being mild anxiety and 33.3% moderate to severe anxiety. Among MGFA Class III patients, 50% reported anxiety symptoms, with severity increasing as the disease progressed. In the MGFA Class IV–V group, 54.5% of patients exhibited anxiety symptoms, with a marked increase in severity compared to the lower classification groups.

As shown in Table 4 (corresponding to Figure 2), although the chi-square test for depression did not reach statistical significance (*χ*^2^ = 11.054, *p* = 0.061), the data revealed a trend suggesting an association between MG severity and depressive symptoms. In the MGFA Class I group, 23.5% of patients had mild to moderate depression symptoms. In the MGFA Class II group, 29.4% of patients reported depressive symptoms, with 35% being mild depression and 61.5% moderate to severe depression. Among MGFA Class III patients, 50% exhibited depressive symptoms, with the majority being moderate to severe. In the MGFA Class IV–V group, the proportion further increased, with 54.5% of patients experiencing moderate to severe depression.

### 3.3 Comparison of the risk of anxiety and depression symptoms between mild and moderate-to-severe MG patients

To further examine the relationship between disease severity and psychological symptoms, patients were stratified into two groups based on MGFA classification: mild MG (MGFA Class I–II) and moderate-to-severe MG (MGFA Class III–V). Data presented in Table 5 compare the prevalence of anxiety and depression symptoms between these two groups.

**Table 5:** Anxiety and depression risk in mild vs. moderate–severe Myasthenia Gravis.

Groups	Number of MG patients		Total	*X* ^2^	*p*-value	OR
	Anxiety	Non-anxiety				
Mild	15	53	68	7.788	0.01*	3.83 (1.45–10.11)
Moderate–severe	13	12	25			

Groups	Number of MG patients		Total	*X* ^2^	*p*-value	OR
	Depression	Non-depression				
Mild	19	49	68	6.286	0.016*	3.28 (1.27–8.49)
Moderate–severe	14	11	25			

^*^p < 0.05 is significant. MG, Myasthenia Gravis; OR, odds ratio.

The results indicated that anxiety symptoms were significantly more prevalent among patients with moderate-to-severe MG. Chi-square analysis demonstrated a statistically significant difference (*χ*^2^ = 7.788, *p* = 0.01), with an odds ratio (OR) of 3.83 (95% CI: 1.45–10.11). This suggests that patients with moderate-to-severe MG were 3.83 times more likely to experience anxiety symptoms than those with mild disease. Similarly, for depressive symptoms, a significant difference was also observed between the two groups (*χ*^2^ = 6.286, *p* = 0.016). The odds ratio for depression was 3.28 (95% CI: 1.27–8.49), indicating that the risk of depression in moderate-to-severe MG patients was 3.28 times that of patients with mild MG.

### 3.4 Regression analyses of potential risk factors for anxiety and depression in MG patients

Univariate logistic regression analyses (Table 6) showed that impaired quality of life (MG-QOL-15, adj *p* < 0.001), reduced social support (SSRS, adj *p* = 0.025), and functional limitations (MG-ADL, adj *p* = 0.025) were significantly associated with anxiety and/or depression after FDR correction. Comorbid conditions showed a nominal association in the raw analysis (*p* = 0.037) but did not survive correction (adj *p* = 0.100). Other variables, including demographic factors, antibody status, thymoma, treatment history, and number of crises, were not significantly related to psychological outcomes.

**Table 6:** MG patients clinical factors associated with anxiety or depression (univariate logistic regression).

Clinical Factors	OR (95% CI)	Raw *p*	FDR adj *p*
Age (years)	0.99 (0.96 ~ 1.02)	0.386	0.480
Gender			
Male			
Female	0.95 (0.39 ~ 2.32)	0.906	0.933
Nationality			
Han			
Others	Uncertainty	Uncertainty	Uncertainty
High school or above			
Yes			
No	1.69 (0.64 ~ 4.43)	0.288	0.380
Married			
Yes			
No	0.64 (0.23 ~ 1.88)	0.412	0.480
Have children			
Yes			
No	0.69 (0.23 ~ 2.07)	0.513	0.580
Separated			
Yes			
No	0.44 (0.11 ~ 1.67)	0.224	0.340
MGFA Clinical Classification (Class V)			
Yes			
No	0.34 (0.06 ~ 1.94)	0.223	0.340
MGFA Clinical Classification			
I			
II	4.20 (0.84 ~ 21.05)	0.081	0.170
III	3.208 (0.83 ~ 12.45)	0.092	0.180
IV–V	0.97 (0.19 ~ 5.03)	0.973	0.973
Antibodies [AChR-Ab (+)]			
Yes			
No	0.81 (0.26 ~ 2.55)	0.718	0.790
Thymic condition (thymoma)			
Yes			
No	1.06 (0.45 ~ 2.47)	0.901	0.933
Time since onset (years)	1.02 (0.96 ~ 1.09)	0.507	0.580
Ever glucocorticoid use			
Yes			
No	0.31 (0.06 ~ 1.55)	0.155	0.260
Ever immunosuppresant use (except glucocorticoid)			
Yes			
No	0.65 (0.28 ~ 1.51)	0.317	0.390
Combined with other diseases			
Yes			
No	0.39 (0.16 ~ 0.95)	0.037*	0.100
Number of MG Crisis	0.84 (0.63 ~ 1.13)	0.245	0.340
MG-ADL score	0.83 (0.73 ~ 0.94)	0.005*	0.025*
MG-QOL-15 score	0.87 (0.82 ~ 0.92)	<0.001*	<0.001*
SSRS score	1.08 (1.02 ~ 1.14)	0.006*	0.025*

Raw *p* = unadjusted *p* value from univariate logistic regression. FDR adj *p* = *p* value adjusted for multiple comparisons using the Benjamini–Hochberg procedure (*q* = 0.05). Statistical significance was defined as FDR adj *p* < 0.05.

MG, myasthenia gravis; HADS, Hospital Anxiety and Depression Scale; HADS-A, HADS—Anxiety; HADS-D, HADS—Depression; AChR, acetylcholine receptor; MGFA, Myasthenia Gravis Foundation of America; MG-ADL, Myasthenia Gravis Activities of Daily Living scale; MG-QOL-15, Myasthenia Gravis Quality of Life 15-item scale; SSRS, Social Support Rating Scale.

Multivariable logistic regression (Table 7) confirmed that lower MG-QOL-15 scores (OR = 0.85, 95% CI: 0.78–0.92, *p* < 0.001) and lower SSRS scores (OR = 1.08, 95% CI: 1.00–1.16, *p* = 0.03) were independent predictors of anxiety and depression. MGFA classification was retained in the model as an indicator of disease burden, although it did not reach statistical significance after adjustment.

**Table 7:** Multivariable logistic regression analysis of clinical factors associated with anxiety and depression in MG patients.

Clinical Factors	OR (95% CI)	*p*
MGFA Clinical Classification		
I		
II	0.96 (0.11 ~ 8.51)	0.97
III	1.22 (0.20 ~ 7.60)	0.82
IV–V	2.06 (0.25 ~ 17.23)	0.50
Combined with other diseases	1.46 (0.41 ~ 5.12)	0.55
MG-ADL score	1.08 (0.88 ~ 1.31)	0.47
MG-QOL-15 score	0.85 (0.78 ~ 0.92)	<0.001
SSRS score	1.08 (1.00 ~ 1.16)	0.03

^*^p < 0.05 is significant.

OR, odds ratio; CI, confidence interval; MGFA, Myasthenia Gravis Foundation of America classification; MG-ADL, Myasthenia Gravis Activities of Daily Living scale; MG-QOL-15, Myasthenia Gravis Quality of Life 15-item scale; SSRS, Social Support Rating Scale.

As a supplementary analysis, multivariable linear regression (Supplementary Table 1) was performed with HADS-A and HADS-D scores modeled as continuous outcomes. The results were consistent with the logistic regression models: MG-QOL-15 remained significantly associated with higher levels of anxiety and depression (*B* = 0.48, 95% CI: 0.36–0.60, *p* < 0.001), while SSRS was inversely associated (*B* = −0.17, 95% CI: −0.29 to −0.04, *p* = 0.008). These findings underscore the robust and independent effects of quality of life and social support on psychological distress in MG patients.

### 3.5 Analysis of the relationship between anxiety and depression symptoms and assessment scales: ROC curve evaluation

**Figure 3:**
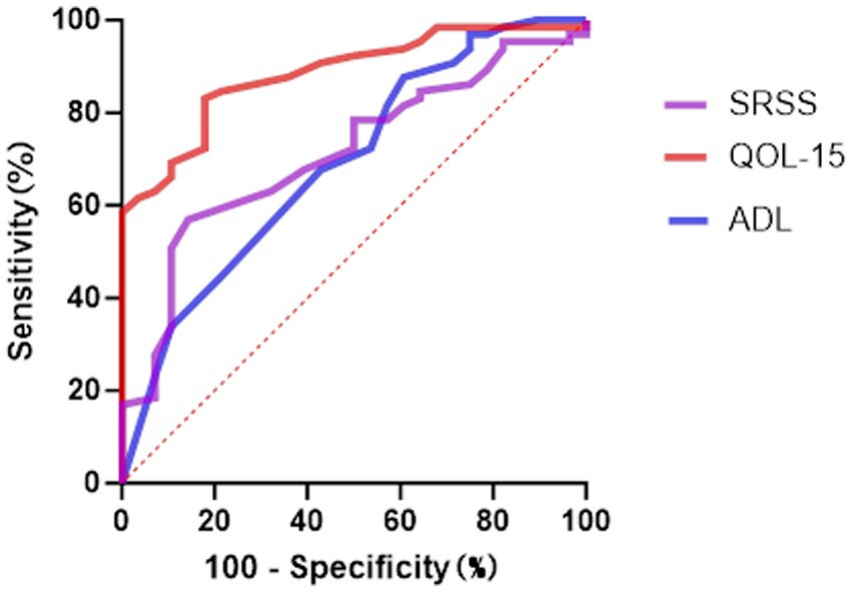
The ROC curve analysis for predicting anxiety symptoms. ROC, Receiver Operating Characteristic; ADL Myasthenia Gravis, Activities of Daily Living; SSRS, Social Support Rating Scale; QOL-15 Myasthenia Gravis, Quality of Life 15-item scale.

**Figure 4:**
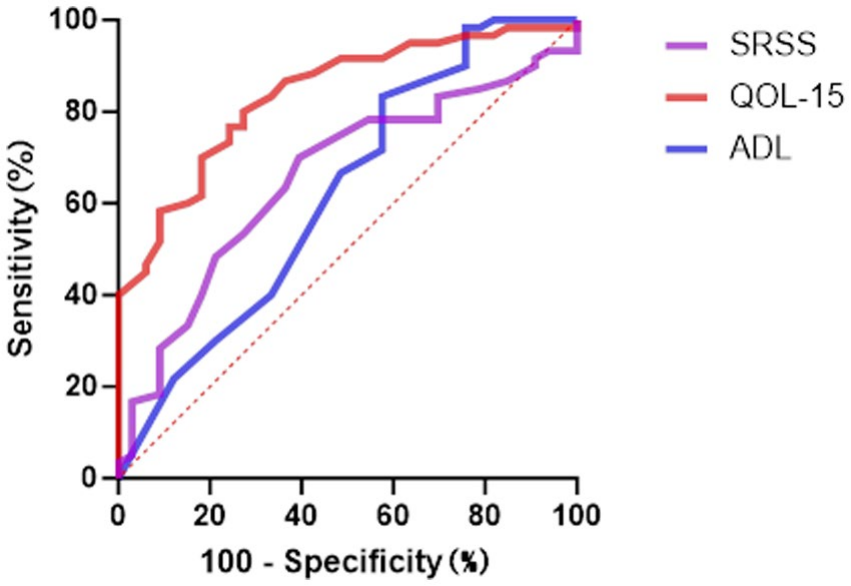
The ROC curve analysis for predicting depressive symptoms. ROC, Receiver Operating Characteristic; ADL Myasthenia Gravis, Activities of Daily Living; SSRS, Social Support Rating Scale; QOL-15 Myasthenia Gravis, Quality of Life 15-item scale.

To evaluate the predictive performance of the scales in screening for anxiety and depression symptoms, ROC curve analyses were conducted for MG-ADL, MG-QOL-15, and SSRS (Table 8, Figures 3–4). In the prediction of anxiety symptoms, MG-QOL-15 performed best, with the highest AUC (0.884, 95% CI: 0.817–0.951, *p* < 0.001). At the cut-off value of 23.5, the scale showed both high sensitivity (0.821) and specificity (0.831), indicating a significant association between reduced quality of life and anxiety symptoms. SSRS also demonstrated acceptable discriminative ability (AUC = 0.712, 95% CI: 0.604–0.821, *p* = 0.001), with sensitivity of 0.569 and specificity of 0.857 at the cut-off of 43.5, reflecting the protective role of social support. MG-ADL showed moderate predictive power (AUC = 0.690, 95% CI: 0.571–0.808, *p* = 0.004), with relatively low sensitivity but higher specificity, suggesting some value in ruling out anxiety.

**Table 8:** ROC analysis for MG-ADL, MG-QOL-15, and SSRS in predicting anxiety and depression.

Scale	Outcome	AUC	SE	*p* value	95% CI lower	95% CI upper	Sensitivity	Specificity	Cut-off value
MG-ADL	Anxiety	0.690	0.060	0.004	0.571	0.808	0.393	0.877	6.5
	Depression	0.622	0.063	0.054	0.498	0.746	0.424	0.833	5.5
MG-QOL-15	Anxiety	0.884	0.034	<0.001	0.817	0.951	0.821	0.831	23.5
	Depression	0.837	0.041	<0.001	0.756	0.918	0.727	0.800	22.5
SSRS	Anxiety	0.712	0.055	0.001	0.604	0.821	0.569	0.857	43.5
	Depression	0.657	0.058	0.013	0.543	0.771	0.700	0.606	41.5

AUC, area under the ROC curve; SE, standard error; CI, confidence interval. Optimal cut-off values were determined using the Youden index. MG-ADL, Myasthenia Gravis Activities of Daily Living scale; MG-QOL-15, Myasthenia Gravis Quality of Life 15-item scale; SSRS, Social Support Rating Scale.

In the prediction of depressive symptoms, results were similar to those for anxiety. MG-QOL-15 again performed best (AUC = 0.837, 95% CI: 0.756–0.918, *p* < 0.001), achieving sensitivity of 0.727 and specificity of 0.800 at the cut-off of 22.5. The discriminative ability of SSRS was relatively limited (AUC = 0.657, 95% CI: 0.543–0.771, *p* = 0.013), while MG-ADL had the lowest predictive value (AUC = 0.622, 95% CI: 0.498–0.746, *p* = 0.054), suggesting only a weak role in screening for depression.

The SSRS also showed reasonable sensitivity, though with lower specificity. MG-ADL exhibited relatively higher specificity in distinguishing non-depressed patients.

## 4 Discussion

This study systematically evaluated the associations between anxiety and depressive symptoms and clinical characteristics in a Chinese cohort of patients with myasthenia gravis (MG). Our findings demonstrated that diminished quality of life (MG-QOL-15) and insufficient social support (SSRS) were most strongly and independently associated with psychological distress. By contrast, traditional measures of disease severity such as MGFA classification and MG-ADL scores, although showing significant correlations in univariate analyses, did not retain independent effects in multivariable models. This pattern suggests that the impact of disease severity on psychological health may be mediated through its consequences for daily functioning and perceived quality of life, rather than exerting a direct influence. In this sense, MGFA classification and MG-ADL remain clinically important indicators of overall disease burden, but their role in mental health outcomes may be indirect and context-dependent. Retaining MGFA classification in the multivariable analysis preserves the interpretability of the model and highlights its value as a background determinant in psychological assessment.

Moreover, ROC curve analyses confirmed the utility of multidimensional scales in screening for psychological distress. MG-QOL-15 exhibited the highest AUC values for both anxiety and depression, underscoring its dual role as a measure of disease impact and as a proxy for mental health status. The SSRS also demonstrated reasonable discriminative ability, reinforcing the protective role of social support. MG-ADL, although less effective in overall discrimination, showed relatively high specificity, suggesting its utility in ruling out low-risk individuals. Together, these findings highlight the need to incorporate multidimensional psychological assessments into routine MG management. ROC-derived thresholds may serve as practical tools for early identification of high-risk patients in clinical settings.

From a psychological perspective, as the disease progresses and functional limitations increase, patients with MG often experience a loss of perceived self-efficacy. In moderate to severe stages, restrictions in daily living activities may lead to feelings of helplessness and emotional distress. Moreover, the chronic and unpredictable nature of MG can foster catastrophizing thoughts, thereby amplifying psychological burden (27). A lack of social support has also been shown to be an important determinant of mental health; individuals with low levels of social support are more prone to feelings of isolation and helplessness, which are associated with heightened anxiety and depressive symptoms (28).

From a mechanistic perspective, previous studies have suggested that chronic inflammation, neurotransmitter imbalance, and hypoxic states may contribute to psychological symptoms in MG, providing a biologically plausible explanation for the observed associations (29–33). However, these hypotheses remain speculative in the absence of direct biological data, and future studies integrating biomarkers with longitudinal follow-up will be necessary to substantiate such pathways. Importantly, MG is not only associated with mood disorders but also with cognitive impairments, including deficits in attention, memory, and executive function, further broadening the scope of psychological burden in this population (10, 34).

Therapeutically, in addition to corticosteroids and conventional immunosuppressants, other interventions such as thymectomy, plasma exchange, intravenous immunoglobulin, and novel biologics (e.g., eculizumab) have been shown to reduce disease activity and improve functional and quality-of-life outcomes (35–39). Although psychiatric outcomes were not primary endpoints in these studies, improvements in physical health and daily functioning are likely to exert beneficial effects on mental health. Future research should explicitly assess the impact of therapeutic strategies on psychological well-being to inform integrated management approaches.

Several limitations should be acknowledged. First, the single-center, cross-sectional design and modest sample size, particularly in patients with severe MG, may limit statistical power and the stability of effect estimates. Second, although we adjusted for age and sex, residual confounding cannot be excluded, such as cumulative medication exposure, psychiatric history, or socioeconomic status. Third, while FDR correction was applied to reduce the risk of type I error in univariate analyses, replication in larger and independent cohorts is needed. Finally, the single-center nature of this cohort may restrict the generalizability of our findings to broader populations.

In conclusion, this study demonstrates that anxiety and depression in MG are strongly associated with reduced quality of life and insufficient social support, with disease severity contributing indirectly through these mediators. These findings underscore the need for routine psychological screening and multidimensional assessment in MG care, and support a holistic management model that integrates physical treatment with mental health and social support interventions to optimize overall outcomes.

## 5 Conclusion

This study systematically evaluated the associations between anxiety and depressive symptoms and clinical characteristics in a Chinese cohort of patients with myasthenia gravis (MG). We found that diminished quality of life and inadequate social support were the strongest correlates of psychological distress, while conventional indicators of disease severity, such as MGFA classification and MG-ADL scores, showed associations in univariate analyses but did not retain independent significance after adjustment.

These findings underscore the importance of incorporating routine psychological assessment and targeted mental health interventions into the comprehensive care of MG patients. Screening instruments such as the MG-QOL-15 and SSRS may provide clinically practical cut-off values to facilitate early identification of high-risk individuals.

Future research should prioritize longitudinal validation to clarify causal pathways, integrate biological and psychosocial markers to explore underlying mechanisms, and assess multimodal interventions—including pharmacological treatment, psychological therapies, and social support programs—to optimize both mental health outcomes and overall quality of life in MG patients.

## Data Availability

The raw data supporting the conclusions of this article will be made available by the authors, without undue reservation.
