# Supplementary material for: Quality of life and social support as key determinants of anxiety and depression in Myasthenia Gravis: evidence from a Chinese cohort
Source: Front Neurol. 2025 Nov 12;16:1670436. doi: 10.3389/fneur.2025.1670436 (PMC12647006; doi:10.3389/fneur.2025.1670436)
Supplement: Supplementary file 1 [file Table_1.docx]

**Supplementary Table 1.** Multivariable linear regression analysis of clinical factors associated with continuous anxiety and depression scores in MG patients

| Clinical factors | B (95%CI) | P |
| --- | --- | --- |
| MGFA Clinical Classification |  |  |
| I |  |  |
| II | 0.07(-1.70~3.89) | 0.44 |
| III | -0.12(-6.49~1.71) | 0.25 |
| IV-V | 0.01(-3.98~4.38) | 0.92 |
| Combined with other diseases | -0.01(-2.37~2.23) | 0.95 |
| MG-ADL score | -0.08(-0.51~0.20) | 0.39 |
| MG-QOL-15 score | 0.48(0.36~0.60) | <0.001 |
| SSRS score | -0.20(-0.29~-0.04) | 0.01 |

*Note:* *P< 0.05 is significant.

*Abbreviations:*B = unstandardized regression coefficient; CI = confidence interval; MGFA = Myasthenia Gravis Foundation of America classification; MG-ADL = Myasthenia Gravis Activities of Daily Living scale; MG-QOL-15 = Myasthenia Gravis Quality of Life 15-item scale; SSRS = Social Support Rating Scale.
